# Evaluation of the effect of surface roughness and modification of implant abutment axial wall on the retention of zirconium oxide copings before and after thermocycling

**DOI:** 10.4317/jced.61175

**Published:** 2024-04-01

**Authors:** Parviz Amini, Maryam Hejazi, Masumeh Taghva, Sina Safari

**Affiliations:** 1 DDS. Prosthodontics Research Center, School of Dentistry, Kerman University of Medical Sciences, Kerman, Iran; 2 DDS, MSc. Department of Prosthodontics, School of Dentistry, Shiraz University of Medical Sciences, Shiraz, Iran; 3 DDS, MSc. Department of Prosthodontics, School of Dentistry, Kerman University of Medical Sciences, Kerman, Iran

## Abstract

**Background:**

The purpose of this study was to determine the effect of one-wall elimination of the abutment and also the surface treatment of the abutment on the retention of cement-retained, implant-supported zirconium oxide copings.

**Material and Methods:**

In this experimental study, four straight abutments were connected to four implant analogs (DIO, UF, Busan, Korea) with 35 Ncm torque. They are mounted vertically in resin blocks. Abutments were prepared as following groups: A) abutment was used in its intact standard form as a control group. B) 4 mm of the flat wall was removed to produce an abutment with 3 walls. C) The abutment surface was abraded with 50 µm AL2O3 powder. D) 4 mm of a flat wall of the abutment was removed, then the abutment surface was abraded with 50 µm AL2O3 airborne particle.10 zirconium oxide copings were made. Samples were cemented with temp bond NE to abutments. The retention of copings was measured before and after incubation using the universal testing machine. T-test, one-way ANOVA, and Post Hoc Tukey Test were used for statistical analysis of data.

**Results:**

In all groups, retention was decreased after thermocycling (*P* ≤0.001). 3 wall abutments had less retention than the control group before thermocycling. A significant difference was detected between 3 wall abutments and 4 walls of sandblasted abutments before thermocycling. After thermocycling, no difference in retention was seen between groups.

**Conclusions:**

Thermocycling significantly reduces the retention of implant-supported ceramic copings. Sandblasting abutments with 50 µm AL2O3 air-borne particles did not increase the retention of ziconium oxide copings which were cemented with temp bond NE. One wall elimination of abutment decreased the retention of zirconia copings.

** Key words:**Retention, crown, abutment, zirconia coping, sandblasting.

## Introduction

A revolution in the treatment of edentulous areas occurred with the introduction of implants (1). In recent years, zirconium oxide ceramic has been introduced, and it improved the esthetic in implant treatments (2).

Zirconia stabilized with yttrium became popular as a suitable material for the base of the prosthetic superstructures on implants. Polycrystalline ceramics present high mechanical properties and esthetic and biological benefits such as reducing the accumulation of plaque. Besides, manufacturing ceramic restorations with CAD / CAM has benefits like high marginal accuracy (3).

Now controversy about the selection of screw-retained or cemented restorations exists (1). Cemented crowns are more popular so more sensitive Patients prefer them due to the lack of screw access holes and more similarities to natural teeth (2,4). Other advantages of cemented implant-supported crowns include Fewer side effects, higher fracture resistance (4), more tight occlusion, appropriate loading along the longitudinal axis of the implant (5), better passive fitness, smaller occlusal table due to the absence of screw access hole, lower cost, more easily clinical practice in a shorter time especially in the posterior of the mouth (3), reducing the probability of porcelain fracture (6), more uniform stress distribution (7). The only significant advantage of screw-retained prosthesis is it’s easier retrieval possibility. The ability to bring out the restorations on the implants is needed in the periodic replacement of prosthetic components, loosening or breaking the abutment screw, the abutment fracture, changing the prosthesis because of an implant failure, and further surgical intervention (8).

According to Goodacre *et al*., retention loss of the crown is the most common mechanical failure in cement-retained implant restorations (9).

The retention of cemented restorations depends on the abutment geometry, surface roughness, and the type of cement (10,11).

Cement selection is one of the important factors controlling the retention of restorations (12-16) choosing more retentive cement like zinc phosphate, polycarboxylate, glass ionomer, and resin-modified glass ionomer can damage the implant fixture, implant abutment, and the screw of abutment or prosthesis if aggressive technique is used to remove the restoration (17). It is recommended to cement all implant-supported restorations with temporary cement to retrieve restoration easily without damage (7). Sometimes temporary cements cannot provide the minimum required retention for implant prostheses so other ways are needed to improve the retention (18).

According to Ajay *et al*. sandblasting and acid etching can improve the retention of Ni-Cr copings made on titanium abutments (19). Oxygen plasma and sandblasting treatment with 50 µm Al2O3 has been shown to enhance the retention of metal copings (20). Furthermore, airborne particle abrasion with 50 µm Al2O3 increased the bond strength between the base abutments and lithium disilicate restorations (21). According to* Kemarly *et al*. mechanical surface treatment( sandblasting or CoJet silicoating) is more important than chemical surface treatment for bond strength of Lithium disilicate copings to ti-base abutments (22).

Implant geometry is another important factor that affects the retention of cement-type implant restorations (23-26). Kian *et al*. found that implant abutments with three walls had more retention than those with two walls (25). However, Derafshi, *et al*. found that three and four wall abutments had the same retention (26).

The effect of sandblasting and the number of implant abutment walls on the retention of zirconia crown is not obvious. This study aims to determine the effect of surface roughness and one-wall elimination of abutment on the retention of cemented zirconia-based implant-supported restorations.

## Material and Methods

Four straight abutments were connected to four implant analogs (DIO, UF, Busan, Korea) with 35 Ncm torque. The abutment diameter and height were 4.5 and 5.5 mm, respectively. Each analog was mounted vertically in resin blocks (Acropars 200, Marlic, Iran) with Surveyor (Mariotti, Bravo, Italy) while the abutment-analog interface was 1 mm above the acrylic surface.

**Figure 1:**
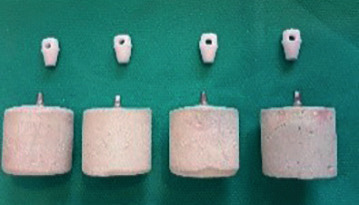
Prepared groups.

Then abutment preparation was done as follows: (Fig. 1)

Group A: intact abutment was considered as a control group. ( 4 walls)

Group B: The 4 mm height of the abutment flat wall was removed with a tapered carbide bur. ( 3 walls) 

Group C: the abutment surface was sandblasted with 50 microns of aluminum oxide abrasive particles at a pressure of 2.5 bar and a duration of 15s from 10 mm distance (Rentfert, Germany). ( 4 walls sandblasted) 

Group D: The 4 mm height of the abutment flat wall was removed with tapered Carbide bur, then the abutment was sandblasted with 50 microns of aluminum oxide abrasive particles with a pressure of 2.5 bar and a duration of 15s from a distance of 10mm. An acrylic resin index was used to achieve the uniform shape of the removal wall in groups B and D ( 3 walls sandblasted).

Each abutment was scanned with the 3D scanner (3 shape D810, Denmark) then, 10 ceramic copings with a loop on the occlusal surface were designed with the specialized software Dental Designer. Zirconium oxide copings (Dental Direkt (ZW), Germany) were made with CAD-CAM (IMes-icore (450i), Germany). 25-micron space was considered for the cement up to 2 mm above the margin.

All copings were sintered at 1200°C for 12 hours after the preparation. The accuracy of copings was checked with silicon disclosing medium (Dentaco, Germany) and marginal fitness was evaluated with visual and tactile methods using a sharp explorer. Temporary restoration (Cavit, Golchai, Iran) was used to seal the abutment screw access hole.

**Figure 2:**
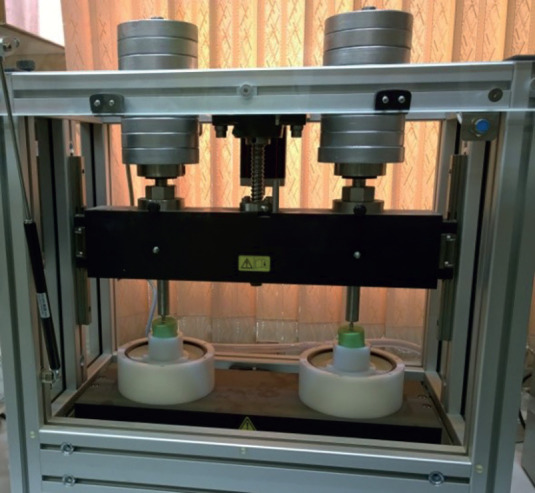
The chewing simulator machine.

Tests were done in two stages before thermocycling and after thermocycling:

 In the first stage, copings were cemented with temp bond NE (Kerr, Italy) according to manufacturer instructions. The cement was mixed at room temperature for 30 seconds by an operator. Cement was gently applied to the copings then according to ADA specification N.96, they were kept under 5 kg force for 10 min in the chewing simulator (SD Mechatronik, Germany) (Fig. 2). After the initial setting, excessive cement was removed with a plastic explorer. After 24h of resting the samples, they were connected from the loop area to the clamp of the Instron universal testing machine (Testometric, M350-10CT, Germany). Vertical tensile forces were applied at 0.5 mm/min cross-head speed parallel to the longitudinal axis of the samples. The force in which the bond failure had happened was recorded in Newton for each sample.

The copings were checked with the stereo microscope to determine the presence or absence of Crack as well as the bond failure mode. The Failure Mode of cement is described in Table 1.

**Table 1:**
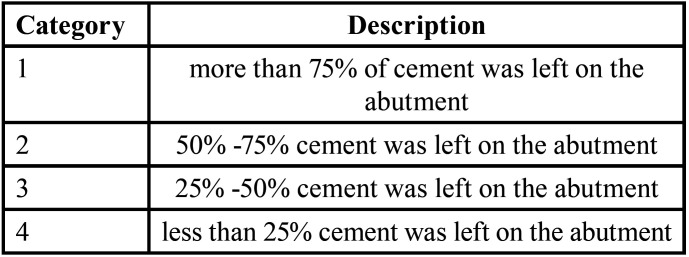
The mode of failure of cement categories.

To perform the second stage of the test, the remaining cement in abutments and copings were cleaned with a plastic explorer and immersed in ultrasonic cleaners (Ultrasonic, Bandelin, Super RK102H, Germany) for 15 minutes. Abutments and copings were washed with water and stored in distilled water for 5 minutes, then they were dried. Finally, the samples were checked visually. After recementation with tempbond NE, the samples were stored in 100% humidity at 37°C for h24. Then the samples were subjected to 1000 thermal cycles, at the temperature 5 to 55°C (TC-300, Iran) with 30 seconds of dwell time. The specimens were dislodged by applying tensile force parallel to the longitudinal axis with the Instron universal testing machine (Testometric, M350-10CT, Germany). The maximum amount of the dislodgement forces was recorded similar to the first stage.

One-way ANOVA, t-test, and Post Hoc Tukey Test were used for statistical analysis of data.

## Results

The mean, standard deviation, minimum and maximum removal force, and significant differences between the two stages of tests are shown in Table 2. The maximum removal force was related to 4 walls of sandblasted groups which were (150.61± 16.09 N) before incubation and thermocycling and (47.35 ±31.17 N) after incubation and thermocycling. Minimum removal force was related to 3 walls abutment group (107.47±25.4 N) before thermocycling and (23.92± 11.72 N) after the incubation and thermocycling). In all groups, retention was decreased after incubation and thermocycling (*P* ≤0.001). 3 wall abutments (groups B and D) had less retention than the control group before incubation and thermocycling (p.value = 0.004 and 0.022 respectively). A significant difference was detected between 3 wall abutments (group B) and 4 walls of sandblasted abutment (group C) before thermocycling. Therefore; before thermocyling 4 walls sandblasted abutment had more retention (P.value = 0.000). After the incubation and thermocycling, no difference was seen between groups.

**Table 2:**
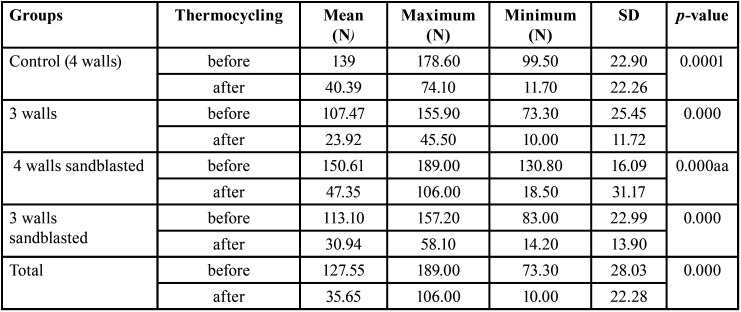
Mean, maximum, minimum, and p-value of groups before and after incubation and thermocycling.

**Figure 3:**
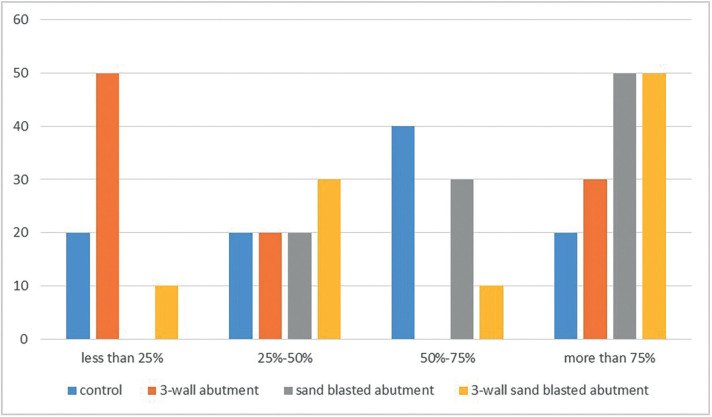
The prevalence of residual cement on abutment in groups before incubation and thermocycling.

**Figure 4:**
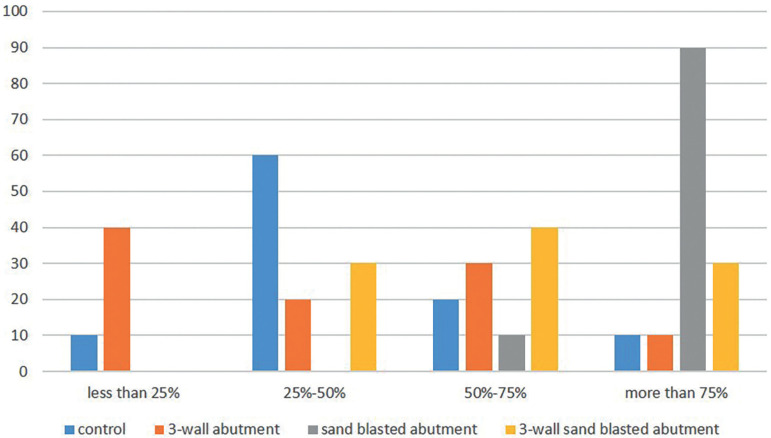
The prevalence of residual cement on abutment in groups after incubation and thermocycling.

The adhesive Failures of cement were observed in all groups. The prevalence of the remaining cement on abutments before and after thermocycling were shown in Figure 3 and 4, (Table 3).

**Table 3:**
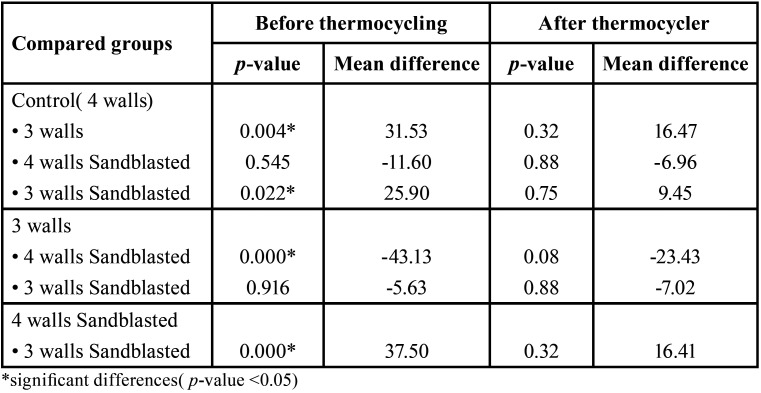
Differences in removal force between groups before and after thermocycling.

## Discussion

In this study, the force required to remove the implant-supported zirconia copings was evaluated before and after the incubation and thermocycling. The study found that the removal force was significantly reduced after incubation and thermocycling.

The current study showed that the removal of an abutment wall as often necessary to modify the angle of the implant body, reduced the retention of zirconia copings compared to the control group before thermocycling. While sandblasting the 3-wall abutment can increase coping retention, this amount is not significant statistically. Abutments of groups 3 and 4 sandblasted with 50 microns of aluminum oxide particles compared to similar non-blasting groups showed a higher average of removal force but it was not statically significant.

After thermocycling, the values of the tensile strength dramatically decreased and modifications of the abutment did not have a significant positive effect on retention.

In Kokubo *et al*.,s study, the effect of sandblasting and thermocycling on the retention of zirconia copings on zirconia abutments using 5 temporary cements was evaluated. The minimum retention was reported for temp bond NE cement in all situations (before and after thermocycling and with or without sandblasting). After thermocycling, the retentive force was significantly decreased in all abutments with or without sandblasting. When temp bond NE was used, sandblasting the abutment did not affect the retention of copings (2). The results are similar to this study. In a study done by Wolfart *et al*., retention of the restorations cemented with some of the cements was affected by sandblasting but the retention of groups that were cemented with free zinc oxide cements was not affected by sandblasting (27). According to Reddy *et al*. the higher surface roughness does not influence the retention values of eugenol-free zinc oxide or TempBond cements. However, they found airborne particle abrasion increased the retention for specimens which was cemented with ImProv (28). Therefore increasing the retention of restoration by increasing surface roughness could be cement-dependent and the low cohesive strength of some cements may inhibit the booster effect of surface roughness on retention (29).

De Campos *et al*. determined the influence of abutment’s surface topography on the retention of single implant-supported metal copings. The research showed that sandblasting with 80-micron aluminum oxide particles and circumferential groove on abutment can increase the retention of metal copings significantly relative to the machined abutments if zinc phosphate cement is used. There was no significant difference between the sandblasted abutment and abutment with groove (30). In the present study, the average removal force in the sandblasted group was more than the control group before and after thermocycling, but statistical differences did not exist probably due to the smaller size of the aluminum oxide particles, different types of copings and different cement.

In Nejatidanesh *et al*, ‘s study, the retention of zirconium oxide implant-supported restorations using different cements was evaluated. All samples abraded with 110 microns of aluminum oxide particles and 9 cement were compared. All samples were incubated for 24 hours and thermocycled for 5,000 cycles, 5-55°Cwith 30 seconds of dwell time. The highest retention was reported for resin cement and the lowest one for temporary and glass ionomer cements. They suggested that temporary cement and glass ionomers are not suiTable for the bonding of single zirconium oxide crowns (4). In the present study, temp bond NE is not recommended in the mouth as a temporary cement for single implant-supported zirconium oxide restoration because of the very low retention force after thermocycling. The selection of cement that does not provide adequate retention can be a source of retentive failure of restoration.

Farzin *et al*. evaluated the effect of the one wall removal of abutment and cement type on implant-supported crown retention. The screw access was filled with cotton and composite resin in the control group and abutments with intact walls. In the experimental group, 4 mm of the height of the flat wall of the abutment was removed, the screw was laid with cotton, and the rest of the way was held open. In this study, the removal force of the 3-wall abutment group was significantly higher than the control group when the temp bond cement was consumed. However, the difference was not observed using Dycal (7). These contrary results to the present study may be related to the present study method in which the screw access was filled with a temporary restoration in all groups and removing a wall of abutment reduced the surface area compared to the control group so the retention was decreased. Another similar study was done by Tan *et al*. in 2012. They concluded that decreasing the number of abutment axial walls from 4 to 3 and from 3 to 2 opposing walls will increase the retention. They did not fill the screw access canal and this can explain the opposite findings to the existing study. Tan *et al*. related these contradictory findings to the rougher internal axial wall and the vent-like performance of screw access canal for cement which facilitates restoration seating (25).

While Sandblasting with 50 microns of aluminum oxide particles did not increase the retention of Zirconium oxide copings significantly, it is recommended to evaluate the effect of sandblasting with different sizes without abutment weakening on the retention of zirconium oxide copings in future studies.

It is also recommended studies evaluate the maximum forces that could transmit to the screw abutments, implant body, and the implant-bone contact while the removal force is applied to cemented restorations without any damage to components of the implants or the least retentive strength required to prevent displacement to easy retrieval of implant-supported restoration. In vitro studies such as this study do not replace clinical ones so their results should be interpreted clinically with caution.

## Conclusions

1) Incubation and thermocycling significantly reduce the retention of implant-supported ceramic copings.

2) Sandblasting abutments with 50 µm AL2O3 air-borne particles did not increase the retention of zirconium oxide copings

3) The retention of the 3-wall abutment group was decreased as compared to the control group and it is not compensated by sandblasting.

4) There was no significant difference between the groups after thermocycling. Since thermocycling reduces significantly the retention of copings, the clinical use of eugenol-free temporary cement (temp bond NE) for single implant-supported zirconia crowns is doubtful.
